# Unraveling the Interactions between Lithium and Twisted Graphene

**DOI:** 10.3390/ma17091941

**Published:** 2024-04-23

**Authors:** Maximo Ramírez, Giorgio De Luca, Lorenzo Caputi

**Affiliations:** 1 Independent Researcher, 87036 Rende, Italy; aquiles011@gmail.com; 2 Research Institute on Membrane Technology (ITM-CNR), c/o University of Calabria, 87036 Rende, Italy; 3 Surface Nanoscience Group, Department of Physics, University of Calabria, 87036 Rende, Italy; lorenzo.caputi@fis.unical.it

**Keywords:** twisted graphene, Li and Li^+^ interaction energies, DFT comparative study, structure-properties relationships

## Abstract

Graphene is undoubtedly the carbon allotrope that has attracted the attention of a myriad of researchers in the last decades more than any other. The interaction of external or intercalated Li and Li^+^ with graphene layers has been the subject of particular attention for its importance in the applications of graphene layers in Lithium Batteries (LiBs). It is well known that lithium atoms and Li^+^ can be found inside and/or outside the double layer of graphene, and the graphene layers are often twisted around its parallel plane to obtain twisted graphene with tuneable properties. Thus, in this research, the interactions between Li and Li^+^ with bilayer graphene and twisted bilayer graphene were investigated by a first-principles density functional theory method, considering the lithium atom and the cation at different symmetry positions and with two different adsorption configurations. Binding energies and equilibrium interlayer distances of filled graphene layers were obtained from the computed potential energy profiles. This work shows that the twisting can regulate the interaction of bilayer graphene with Li and Li^+^. The binding energies of Li^+^ systematically increase from bilayer graphene to twisted graphene regardless of twisted angles, while for lithium atoms, the binding energies decrease or remain substantially unchanged depending on the twist angles. This suggests a higher adsorption capacity of twisted graphene towards Li^+^, which is important for designing twisted graphene-based material for LiB anode coating. Furthermore, when the Li or Li^+^ is intercalated between two graphene layers, the equilibrium interlayer distances in the twisted layers increase compared to the unrotated bilayer, and the relaxation is more significant for Li^+^ with respect to Li. This suggests that the twisted graphene can better accommodate the cation in agreement with the above result. The outcomes of this research pave the way for the study of the selective properties of twisted graphene.

## 1. Introduction

Carbon is one of the most abundant elements on Earth, a component of all known life, and the basis of organic chemistry [1]. Due to its bonding properties, carbon can form several allotropes. From these systems, graphene, since it was isolated by Novoselov et al. in 2004 [2], is without a doubt what has attracted the attention of a myriad of researchers around the world more than any other allotropes thanks to its ballistic electronic transport, thermal conductivity, surface area properties, and large abundance of the starting material for its synthesis [3]. Graphene is a two-dimensional (2D) material, which contains two non-equivalent atoms per unit cell, arranged in a hexagonal lattice. It has been used in a wide range of applications, especially in electrochemical generation and energy storage, solar cells [4], fuel cells [5] and lithium-ion batteries (LiBs) [6]. Wei et al. [7] have experimentally found that graphene has potential in electrochemical energy storage due to its fully accessible surface and unique surface chemistry. Moreover, thermal and absorption properties have been studied, as well as defect formation, graphene doping, and graphene modeling [8,9,10]. Also, graphene applications are expanding to other areas such as desalination, water purification, and biosensors [11,12]. Table 1 summarizes some key properties of graphene, bilayer graphene, and few-layer graphene, i.e., two-dimensional material consisting of 3–10 well-defined stacked graphene layers.

A phenomenon that has attracted considerable attention is the interaction of Li and Li^+^ with graphene layers especially as an application in rechargeable LiBs, which can be found in a wide range of devices from portable electronic devices to electric vehicles. In LiBs, the anode is coated with graphite in contact with a solid electrolyte interface. Graphite is loaded with lithium atoms which yield electrons to the graphite and then to the current collector transforming into Li^+^, which diffuses to the cathode. Although the complex processes that take place in the SEI/graphite system are not well known, the difference in binding energies between Li and Li^+^ with the graphitic layers is certainly a key factor since it is connected with the absorbent capacity of graphitic layers and the electron transfer to the collector and, therefore, with the efficiency of the LiBs. The graphite-based anode shows several limitations such as reduced spacing between layers and different coordination with ions. Furthermore, the preparation of graphite anodes requires polymer binders to achieve good adhesion to the current collector. Laser-induced graphene has proven to be a promising material for improving the performance of LiBs. Alhajji et al. [13] recently showed the important characteristics of laser-induced graphene, such as an increase in ion diffusion, an increase in active sites for efficient adsorption of mobile ions, and an efficient current collector for lithium-metal batteries.

It is well known that lithium atoms and cations can be found inside and/or outside two graphene layers; thus, information on the interaction of Li/Li^+^ with them is quite important. When two single-layer graphenes are stacked vertically, bilayer graphene is obtained; then, if the in-plane angle formed by the two layers is modified, it results in the so-called twisted bilayer graphene [14]. Thus, a double-layer graphene is often twisted around its parallel plane to obtain a twisted graphene and control some properties [15]. Adjusting the twisted angle, electronic properties like chiral tunneling [16], band structure [17], and Fermi velocity of the Dirac electrons [18], among other features [18,19,20,21,22], can be regulated. Twisted graphene is therefore an interesting material that deserves attention in relation to its new characteristics that can be exploited in novel LiB anode coatings. Thus, understanding the Li/Li^+^ interaction with twisted graphene, at an atomic scale, is important for various applications. For instance, many membrane-based ion separation technologies require high monovalent cation selectivity; nevertheless, the available ion exchange membranes usually show a limited specific selectivity towards monovalent ions as required for the Li^+^ recovery from brine feeds. A characterization of the interactions between Li^+^ and twisted graphene is hence important also for a selective recovery of lithium, providing an alternative to classical cation exchange membranes.

Because of its relative cost and accuracy with respect to in-situ experiments, computational simulation methods, in the framework of first-principles Density Functional Theory (DFT), have been employed to study materials at the atomic scale in order to characterize the interaction strength among single-layer Li and graphene, double-layer graphene, and twisted graphene. For example, Robledo et al. [21] found through DFT calculations that the binding of the lithium species in these materials is completely different from that observed in pristine graphite. Also, Zhou et al. [23] have performed a DFT study on the energy stability of lithium adsorption on a single graphene layer with point defects. Larson et al. [24] obtained energetic lithium intercalation for different twist angles using DFT calculations for angles from 2.45° to 7.34°—the first being the larger cell and the second the smaller cell. As the twist angle of the bilayer decreases, the supercell scales to large numbers of atoms, becoming computationally expensive. In the same way, Uchida et al. [18] have accomplished extensive DFT calculations on twisted graphene and they found that for a twist angle of 30°, the two layers are decoupled, while on the other hand, other twist angles near 0° and 60° are strongly coupled. Although there are several works on twisted graphene layers, they are mostly devoted to their electronic properties, while to our knowledge, a comparative study of the adsorption capacity of twisted graphene towards Li and Li^+^ is absent. As highlighted above, extensive research is underway on graphene-based materials for the preparation of electrodes. In this context, the study of twisted graphene takes on particular importance for the development of new classes of carbon materials.

In particular, the adsorption capacity is an important property that allows us to understand whether BLG and graphene-based twisted materials could be used for the preparation of the anodes in LiBs, hence, accelerating their application. The binding energies allow us to obtain information on the adsorption capacity of these materials, providing information on this new material that is very difficult to obtain experimentally. Thus, the novelty of this research study consists in evaluating the above property as a function of the twisted angle of graphene layers, which has not yet been evaluated. Furthermore, from a theoretical point of view, this work introduces a different computational approach for the calculation of binding energies, overcoming some limitations associated with the calculation of the binding energies using the traditional method.

The main aim of this research is to study the interaction energy between Li/Li^+^ and bilayer graphene, as well as twisted bilayer, by using a first-principles DFT approach. This investigation will analyze the interaction energy as a function of the twist angles of the graphene bilayer. Specifically, the potential energy profiles of Li and Li^+^ will be calculated by positioning the atom and cation on double-layer and twisted-bilayer models, and then binding energies (BEs) will be calculated from the potential energy profiles. A comparison of the BEs will be carried out to understand the effect of the twisted layers. Moreover, the potential energy profiles will also be used to evaluate the interlayer equilibrium distances, placing Li and Li^+^ inside the double-layer graphene and twisted-bilayer models.

## 2. Methods and Models

### 2.1. Single Layer, Bilayer, and Twisted Bilayer Models

**Figure 1:**
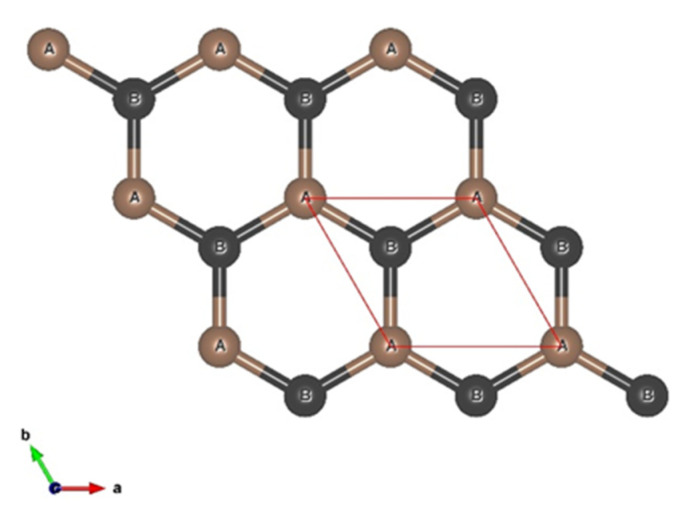
Single-layer graphene cell used in the calculation. The red color rhombus represents the unit cell.

Graphene is a two-dimensional (2D) material formed by two non-equivalent atoms per unit cell arranged in a hexagonal lattice; these atoms are commonly labeled A and B, and represent the sub-lattice, as shown in Figure 1, with a lattice constant a ≈ 2.46 Å, which defines the lattice vectors, and the carbon–carbon distance is 1.42 Å.

Single-layer graphene (SLG), bilayer (BLG), and twisted bilayer (t-BLG) structures were constructed using the 3D visualization program VESTA [20], applying symmetry operation to the unit cell shown in Figure 1. In general, using the transformation matrix (**P**), the lattice vectors (a; b; c) will transform into new lattice vectors (a′; b′; c′) in the following way:

(1) $$\;\left(a^{\prime },b^{\prime },c^{\prime }\right)=\left(a,b,c\right)P\;\;=\left(a,b,c\right)\left(\begin{array}{ccc} P_{11} & P_{12} & P_{13}\\ P_{21} & P_{22} & P_{23}\\ P_{31} & P_{32} & P_{33} \end{array}\right)$$

This is a 3D transformation, but for 2D systems, it can be maintained with an invariant setting: P_33_ = 1 and P_13_ = P_23_ = P_31_ = P_32_ = 0 and the other P_mn_ elements can be parametrized with a couple of integers; thus, the transformation matrix becomes [3]:

(2) $$P=\left(\begin{array}{ccc} m & n & 0\\ -n & m+n & 0\\ 0 & 0 & 1 \end{array}\right)$$

All the periodic models used in the work were designed using the lattice vectors of the graphene unit cell. In detail, we built an SLG, and then two equal SLGs were stacked to obtain the BLG using the experimental interlayer distance. For the special case of t-BLG, we built two layers separately with a specific pair of integers using a (m, n) couple for one layer and (n, m) for the other one with m, n ≠ 0, applying the transformation matrix (2). Then, the two layers were again stacked, remembering that periodicity is maintained only for some twist angles associated with (m, n) couples, as explained below.

Layers of the t-BLG will be commensurate which means that the lattice vectors of both twisted layers must be coincident. In the twisted graphene, only some values of the twist angle retain the overall system periodicity. As suggested in the work of Uchida et al. [14], the twisted angle (*θ*) that allows system periodicity, the number of atoms per cell, (N_atom_) and the cell size (L_cell_) can be calculated according the following equations:

(3) $$cos\theta =\frac{n^{2}+4nm+m^{2}}{2\left(n^{2}+nm+m^{2}\right)}$$

(4) $$L_{cell}=a\left(n^{2}+nm+m^{2}\right)$$

(5) $$N_{atom}=4\left(n^{2}+nm+m^{2}\right)$$

Note that each t-BLG structure is labeled by a pair of integers (m, n) used in the transformation matrix (2). The twist angles used in this research that satisfy the above constraint are summarized in Table 2.

**Figure 2:**
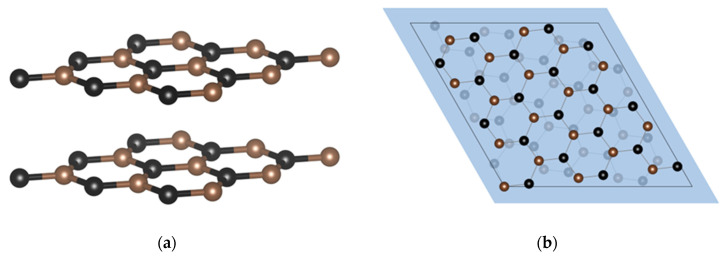
Side view of BLG (**a**) and top view t-BLG (**b**) with twist angle θ = 13.17°.

The BLG structure is composed of 36 atoms/cell, two times the number of atoms of the SLG cell, and has a cell size of 7.38 Å, as shown in Figure 1. In Figure 2, a side view of the bilayer graphene and a top view of the twisted bilayer graphene are shown.

Since the aim of this work is to study the interactions between Li/Li^+^ and twisted graphene, 2D slab periodic systems were used to model the target surfaces. In detail, once we built the SLG, BLG, and t-BLG models, we added a vacuum space between the slabs along the z coordinate perpendicular to the graphene surfaces of 21 Å, obtaining the final simulation cell, where the outermost layer of the slab does not interact with the lower one in the subsequent one.

**Figure 3:**
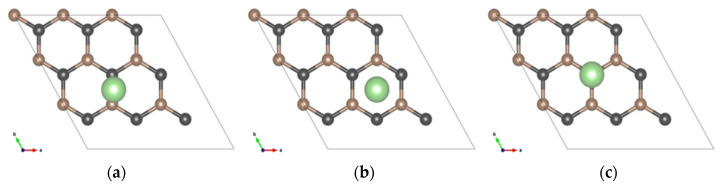
SLG structures for the Li/Li^+^ three absorption sites: (**a**) bridge (B), (**b**) hollow (H), (**c**) top (T).

Moreover, three symmetry adsorption sites for Li/Li^+^ were considered in the calculations; in Figure 3, they are shown for the SLG system.

**Figure 4:**
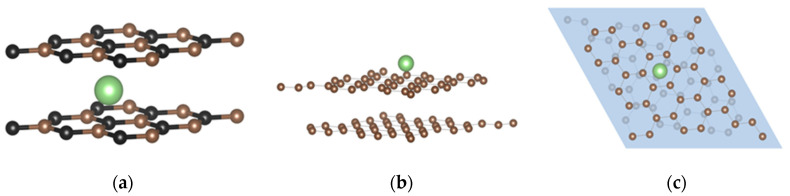
Bilayer graphene structures: (**a**) BLG inside (sandwich) configuration with Li in the bridge side, (**b**) side view of the t-BLG with Li on the hollow site in outside configuration, and (**c**) top view of t-BLG with Li atom on the hollow site in outside configuration.

The lithium atom or cation can be found outside or inside the BLG and t-BLG slabs. Thus, two configurations were considered in the work. Figure 4a shows the BLG structure with lithium between the two layers, named inside or sandwich configuration, while the t-BLG structure with the lithium atom above the slab model (outside configuration) is shown in Figure 4b. Finally, Figure 4c shows the t-BLG with Li in the outside configuration; it is worth noting that the hollow site in the top layer differs from that in the bottom layer.

### 2.2. Computational Approach

Periodic spin-polarized DFT calculations were performed using the Northwest Computational Chemistry Package (NWChem 7.2.2), using the plane-wave (NWPW) module [25,26]. The exchange-correlation contribution of the total energy functional is treated by the Perdew–Burke–Ernzerhof functional (PBE96, White and Bird parameterization) [27]. Plane-wave basis sets were used considering valence electrons only, and the core electrons are described by the norm-conserving pseudo-potential (Hamann pseudopotential) [28]. A quasi-Newton method, limited-memory Broyden–Fletcher–Goldfarb–Shanno algorithm (LMBFGS), was used to minimize the self-consistent energy; the doublet multiplicity of the Li atom was considered when interacting with SLG, BLG, and tBLG slabs. The energy cut-off of the plane wave basis set is set to 60 a.u. while the convergence criterion for the SCF energy difference is 1.0 × 10^−5^ a.u. The periodic calculations used a 3 × 3 × 1 k-point grid in the Brillouin zone.

**Figure 5:**
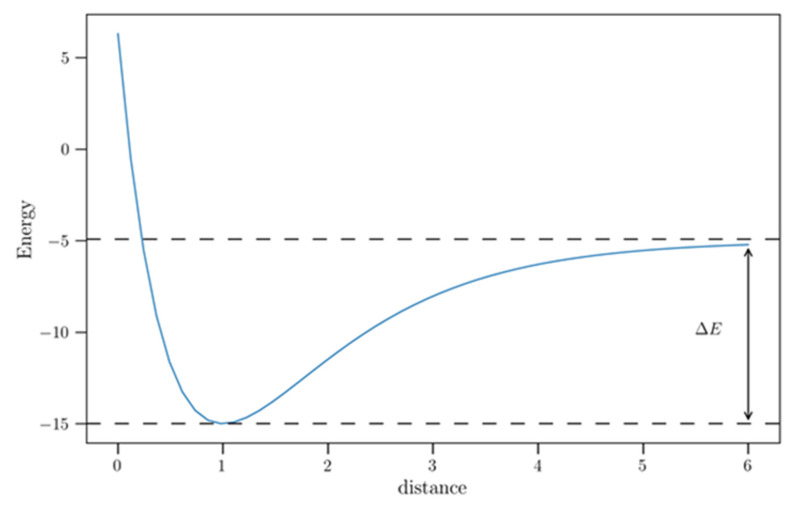
Schematic Morse potential used to evaluate the binding energies of Li and Li^+^ on SLG and BLG in outside configuration. The distance corresponds to the distance between Li or Li^+^ and the adsorption sites.

For the outside configurations, the distance between the two BLG and t-BLG layers was fixed to the experimental value, and then, the total energy was evaluated as a function of the distance between the Li or Li^+^ and surfaces, i.e., the potential energy profile was evaluated for the three adsorption sites. In addition, for the SLG-Li/Li^+^ or BLG-Li/Li^+^ adducts, the potential energy profiles were fitted to an interatomic Morse potential [23]:

*E*(*r*) = *D_e_*(*e*^−2*a*(*r*−*re*)^ − 2*e*^−*a*(*r*−*re*)^) (6)

where *r* is the Li or Li^+^ distance, perpendicular to the adsorption sites, *D_e_* is the equilibrium binding energy at the equilibrium distance *r_e_*, and *a* represents the width of the potential (small value produces depth well). Figure 5 shows a schematic representation of the Morse potential used to fit the computed potential energy profiles.

Then, ∆E was obtained by calculating the difference between the energy of the aforementioned adduct and that corresponding to the energy sum of the non-interacting fragments. The energy of the adduct is calculated considering Li or Li^+^ at the equilibrium distance, i.e., at the minimum of the potential energy curve, while the sum of the energies of the non-interacting fragments can be approximated to the asymptote value of the potential energy. Strictly, to obtain the last energy, the distance between the surface and Li/Li^+^ should be considered infinite. However, for the SLG-Li/Li^+^ or BLG-Li/Li^+^ systems, we considered the asymptote value (plateau) of the Morse potential used to fit the computed potential energy profiles up to 6 Å. We consider the energy of the plateau a good approximation for the sum of the energies of the non-interacting fragments.

In general, ∆E is calculated considering three separate periodic systems according to Equation (7):

(7) $$∆E=E_{slab-Li}-E_{slab}-E_{Li}$$

where $E_{slab-Li}$ is the energy of the adduct, while $E_{slab}$ and $E_{Li}$ are the energies of the surface and Li or Li^+^, respectively. Nevertheless, the calculation of ∆E using Equation (7) involves a correction due to the phase difference of the two subunits; furthermore, the periodic calculation of the energy of the isolate Li^+^ cation, using a pseudo potential, diverges. The calculation of the binding or adsorption energies from the potential energy profiles, as carried out in this work, allows us to overcome these issues. This procedure also allows us to calculate the vibrational modes of the slab-Li/Li^+^ systems, obtaining the corresponding Gibbs free energy of the system more easily [29,30].

Regarding the three adsorption sites considered in the calculations of the potential energy profiles. It should be noted that for the bridge site in the SLG, we found a slight discrepancy between the geometry obtained from the minimum of the potential profile and that obtained by the optimization of the SLG–Li system. Instead, no marked differences were found between the optimized geometries, corresponding to the hollow and top sites of SLG-Li/Li^+^, and those taken from the minima of the potential energy profiles. The optimizations of the geometries were carried out using the Driver geometry optimizer of NWChem. The carbon and Li/Li^+^ atoms in the supercell were fully relaxed during structural optimization using the default thresholds of the Driver module.

## 3. Results and Discussion

### 3.1. Potential Energy and Binding Energy for SGL and BGL

**Figure 6:**
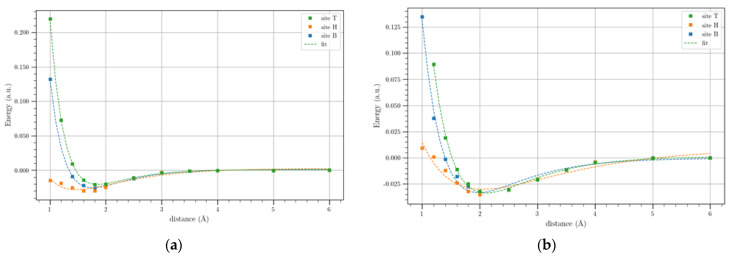
Li (**a**) and Li^+^ (**b**) potential profiles referring to SGL for the three symmetry sites (T, H, and B). The solid lines show the Morse-based potentials.

Figure 6 shows the potential energy profiles of Li and Li^+^ on the SGL for the three analyzed adsorption sites. We observe that the hollow adsorption site is slightly more stable than the others, although the differences are minimal. The Morse-based curve fits the calculated values of Li potential energies very well, while for Li^+^, the fit is still good although to a lesser extent compared to that of Li; in any case, after 6 Å, the profiles reach plateau values.

The binding energies evaluated as the difference between the energy of the SLG-Li/Li^+^ and that of the plateau value, as obtained from the Morse-based curve fitting, are shown in Table 3.

From this Table, we note that the H site provides the larger binding energies for Li and Li^+^ i.e., 0.82 and 0.96 eV, respectively, although the differences with the other BEs are not very large. The equilibrium distance, corresponding to the minimum of the potential energies for the three adsorption sites, was equal, at least for the first digit. This is due to the fact that the difference between the ∆E values is very small, as shown in Table 3, which means that the interactions between SLG and Li^+^ do not change significantly for the three sites, obtaining equal equilibrium distances.

**Figure 7:**
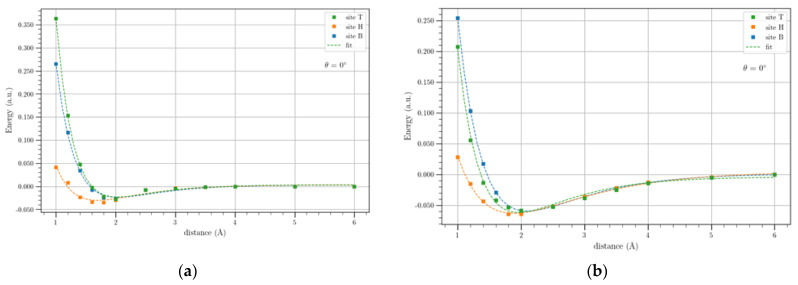
Li (**a**) and Li^+^ (**b**) potential energy profiles on the BLG for the symmetry three sites (T, H, B) in outside configuration. The solid lines show the Morse-based potentials.

Figure 7 shows the potential energy profiles of Li and Li^+^ on the BGL, i.e., for a twist angle of 0°, for the three-symmetry adsorption sites.

As for SLG-Li/Li^+^, the Morse-based potentials fit the DFT calculated values very well, and a stable plateau after 6 Å is reached again. The potential energy profiles for BLG-Li/Li^+^ systems still show the H sites as the more stable compared to the other adsorption sites, providing the same plateau values. For the BLG-Li^+^ systems, the potential curves referring to the H and B sites show equal plateau energies, but for the T site, the potential energy reaches the plateau at higher energy values. It is well known that cations can form stable non-covalent bonds with the benzene ring in which the latter is the electron donor, while the cations are considered acceptors; these bonds are called cation–π interactions. Therefore, it is plausible that the hollow site is the most appropriate position because, in this site, Li^+^ as well as Li can form an optimal interaction compared to other sites.

In Table 4, the binding energies, evaluated as the difference between the energy of the adducts and that of the plateau values, are reported. As expected from the curves in Figure 7, the binding energy of Li and Li^+^ in the H site is higher than the BE referring to the other sites. The equilibrium distance, corresponding to the B and T sites in the BLG-Li/Li^+^ system, was equal, at least for the first digit, as for the case of SLG-Li^+^, because the corresponding binding energies are very similar, as highlighted above. Since the adsorption energies are equal to the binding energies with opposite sing, the value in Table 4 highlights that the H site is the most probable site for the adsorption of Li and Li^+^, even for the BLG, although the differences among the Bes in the case of the BLG-Li^+^ are not very large.

To confirm these preliminary results and verify the reliability of the potential energy approach in the BE calculation, BLG structures, referring to the minima of the Li potential energy, were considered, and the ∆E values were calculated using the traditional Equation (7). Hence, the binding energies, obtained from this equation, were compared with values reported in Table 4, obtained from the Morse-based fit approach.

We found a satisfactory agreement among the binding energies calculated by the two different approaches; this allows us to use the potential energy approach for the calculation of the BEs of the twisted graphene.

### 3.2. Potential Energy and Binding Energy for t-BGL

Regarding t-BGL systems, we again find that in the outside configuration, the hollow side is the most probable site for the Li and Li^+^ adsorption, as reported in Figure S1 (Supplementary Materials); although, for twist angles of 13.17° and 21.79°, the Li potential energies do not show minima up to 6 Å. Then, for the twisted systems, we explored the potential energy curves at distances greater than 6 Å to verify the reliability of the plateaux energy to be used in the calculation of binding energies.

**Figure 8:**
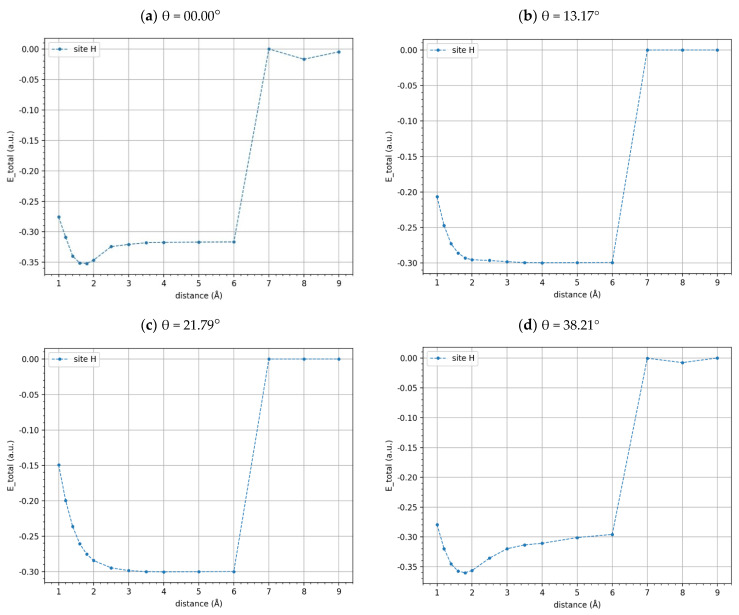
Potential energy profiles of Li adsorbed on BLG and t-BLG, normalized with respect to the energy values of the plateaux for twist angles of 0° (**a**), 13.17° (**b**), 21.79° (**c**), and 38.21° (**d**) (outside configuration).

**Figure 9:**
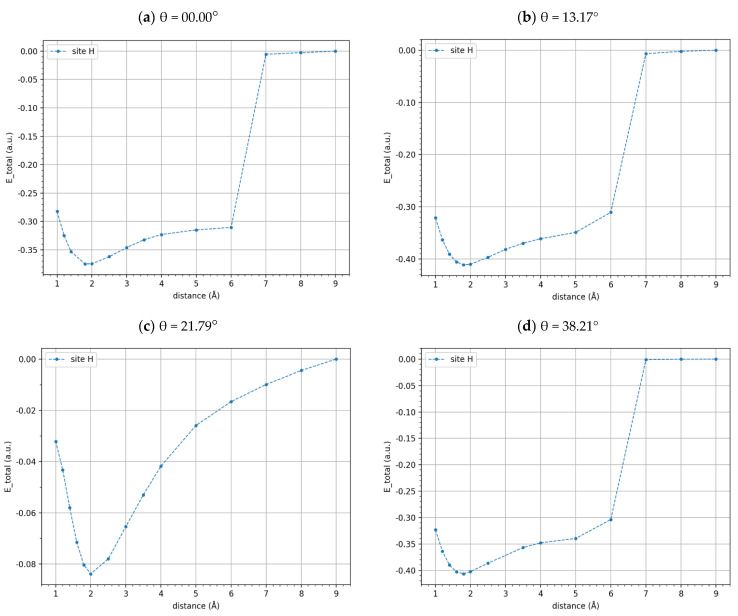
Potential energy profiles of Li^+^ adsorbed on BLG and t-BLG, normalized with respect to the energy values of the plateaux for twist angles of 0° (**a**), 13.17° (**b**), 21.79° (**c**), and 38.21° (**d**) (outside configuration).

**Figure 10:**
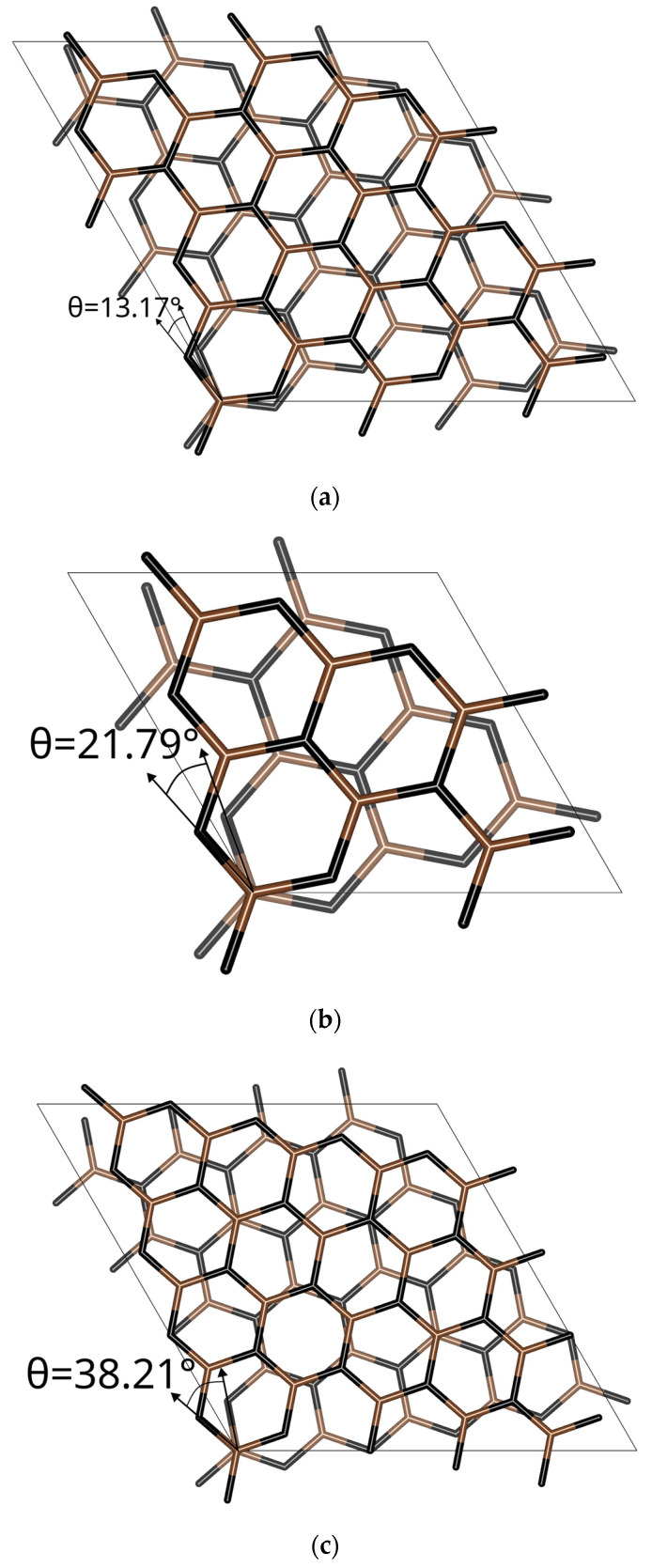
Supercell structures of the different twisted bilayer graphene, corresponding to 13.17° (**a**), 21.79° (**b**), and 38.21° (**c**) used for the calculation of the corresponding potential energy profiles of Li and Li^+^ shown in Figure 8 and Figure 9.

The potential energy curves, normalized with respect to the energy of the plateau values, were shown in Figure 8 and Figure 9 for the t-BGL-Li and t-BGL-Li^+^ systems, respectively. In addition, the structures of the cells showing the two layers of twisted graphene have been reported in Figure 10 for the three different twist angles. The twisted bilayer structures shown in this figure have been used to calculate the potential energy curves shown in Figure 8 and Figure 9, i.e., the potential energy profiles were calculated for each of these periodic structures. Then, the values of the plateau and that of the minimum of these profiles were derived to evaluate the binding energies of Li and Li^+^. From the analysis of the structures shown in Figure 10, we note that for the twist angle of 38.21°, a particular overlap of the phenyl rings appears, generating a rosette shape.

From the analysis of Figure 8 and Figure 9, we note that a systematic and sharp increase in the potential energies between 6 Å and 7 Å is obtained; subsequently, the potential energies stabilize again. As a result, the binding energies of Li and Li^+^ in these systems were calculated as the difference between the energies of the minima and those of the plateaux values reached after 6 Å, which were set equal to zero. It needs to be noted that continuing the plot of the potential energy beyond 10 Å does not make much sense because the Li and Li^+^ at distances greater than 1 nm can be considered non-interacting with the slabs. It is worth noting that the potential energy profiles after 6 Å cannot be fitted by the Morse curve, but this does not affect the calculation of the binding energies because stable energy values after 6 Å were found up to 10 Å.

Analyzing the energy profiles of the t-BLG-Li for twist angles of 13.17° and 21.79° (Figure 8), we observe that a clear minimum was not found; thus, in these cases, we used the value of 0.30 a.u. in the binding energies calculation, which corresponds to the minimum energy value found. Regarding the potential energy profile of the t-BLG-Li^+^ for the twist angle of 21.79° (Figure 9c), we did not find a stable energy value after 6 Å up to 10 Å, even if we did not observe the systematic and sharp increase in potential energies as for the other systems. In this case, we were unable to derive a plateau value from this profile, and as a result, we could not calculate the corresponding binding energy, although this energy potential profile could be fitted with a Morse-based curve.

The binding energies, calculated using the minima and plateau values of the potential energy curves, are reported in Table 5 and Table 6. We find that the binding energies of the Li^+^, interacting with the BLG and t-BLG, are higher than those of Li. This is plausible because the Li^+^ cation can form ion–π interactions with the delocalized orbitals of the slabs, resulting in higher BEs. Furthermore, we observe that the effect of some twist angles on the Li binding energies is more pronounced than the effect found on Li^+^ BEs. This may be due to the fact that the BLG-Li and t-BLG-Li are open-shell systems, unlike the Li^+^ systems, which are closed shells; thus, for the slabs-Li, the binding energies are more affected by the change in the graphene layer alignment. It is important to emphasize that the twisting of the graphene layers causes an increase in the binding energies of Li^+^ regardless of the angle value, specifically passing from a BLG (twist angle 0) to twisted bilayers with 13.17° or 38.21°, respectively; we obtained an increase in binding energy of 1 eV. On the contrary, the binding energies of Li decrease or remain constant depending on the twist angle considered. In detail, for 13.17° and 21.79°, we observe a decrease in the binding energies compared to the BLG of 1.39 eV, while for an angle of 38.21°, a slightly higher BE is obtained with an increase of 0.27 eV. Overall, for the Li^+^ cation, we obtain a clear increase in binding energies, rotating the graphene layers independent of the twist angle.

The values in Table 5 and Table 6 indicate that twisted graphene provides greater Li^+^ adsorption compared to non-twisted layers. This would increase the adsorption capacity of the twisted graphene-covered anode towards Li^+^ in LiBs. Furthermore, this result paves the way for the study of the selective properties of twisted graphene. In fact, if the increase in the adsorption capacity of the twisted graphene is confirmed with respect to cations such as Na^+^, K^+^, and Mg^2+^, this would be important for the selective separations of lithium from brine feeds.

**Figure 11:**
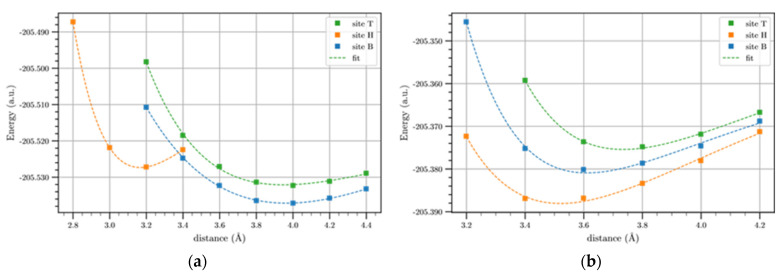
Potential energy profiles of Li (**a**) and Li^+^ (**b**) as a function of the BLG interlayer distance for the symmetry three sites (T, H, B) in the inside configuration. The solid lines are shown for eye guidance.

In addition to the outside configuration, we also analyzed the interactions of Li/L^+^ with BLG and t-BLG slabs in the inside configuration (sandwich). For this configuration, the potential energy profiles of the BLG system for the three symmetry adsorption sites are shown in Figure 11. It is important to note that, in this figure, the interaction energy of the adsorbates has been plotted against the interlayer distances of the slabs.

As a result, the binding energies were not calculated for the inside configuration since separating the two layers until finding a stable value of the potential energy for this configuration does not make physical sense, so the Morse-based potential fit was not carried out in this case. Indeed, for these systems, we focused on finding the equilibrium interlayer distances, corresponding to the minima values of the potential energy profiles.

**Figure 12:**
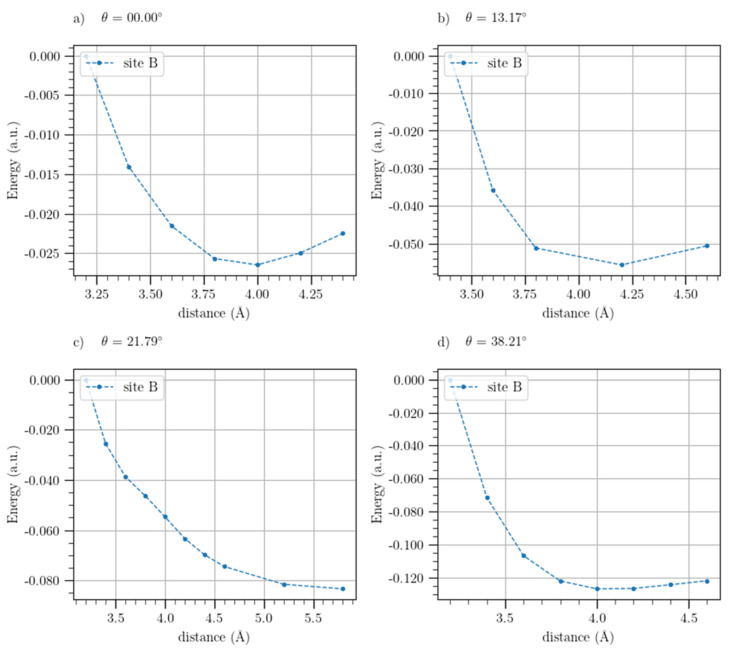
Potential energy of Li as a function of the BLG and t-BLG interlayer distance for twist angles 0° (**a**), 13.17° (**b**), 21.79° (**c**), and 38.21° (**d**) for the bridge site in the inside configuration.

**Figure 13:**
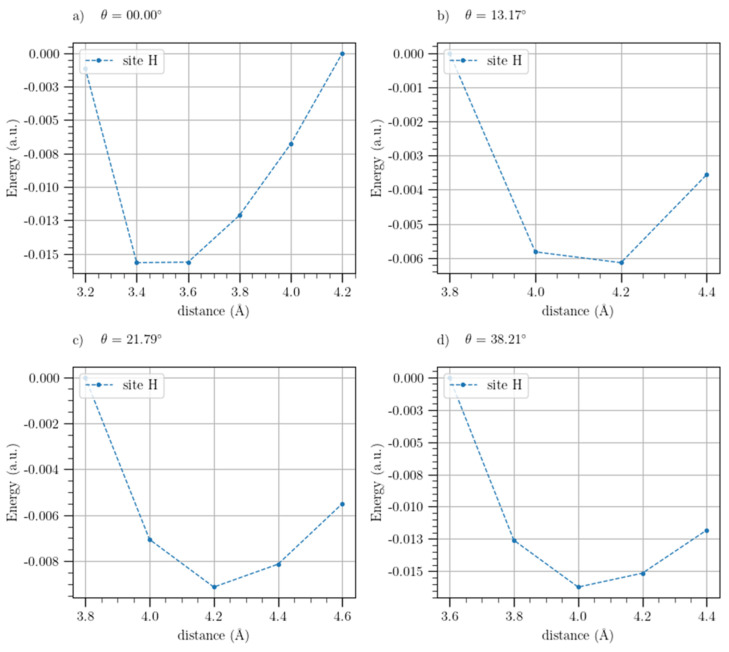
Potential energy of Li^+^ as a function of the BLG and t-BLG interlayer distance for twist angles 0° (**a**), 13.17° (**b**), 21.79° (**c**), and 38.21° (**d**) for the hollow site in the inside configuration.

From the potential energy curves shown in Figure 11, we note that the bridge site is the most stable for Li adsorption, while for Li^+^, the hollow results in the more stable site. Furthermore, in Figure S2 of Supplementary Materials, we also report the potential energy profiles for the t-BLG systems of each adsorption site. From these profiles, it is confirmed that also for the twisted systems, the bridge and hollow are the most stable sites for the adsorption of Li and Li^+^, respectively. Thus, we have shown in Figure 12 and Figure 13 the potential energy curves for these two sites.

According to Figure 12c, the t-BLG-Li potential energy does not provide a minimum value for a twist angle of 21.79°; thus, for this angle, we did not succeed in extracting an equilibrium distance.

In Table 7, we have finally reported the equilibrium distances corresponding to the minima of the potential energy curves.

From Table 7, it can be seen that the insertion of Li in the BGL causes a systematic increase in the experimental interlayer distance of the graphene, whereas the insertion of the Li^+^ causes a reduction in this value. Comparing the values of the equilibrium distances obtained from the computed potential energies profiles, we also observe that the rotation of the graphene layers produces an increase in the equilibrium distances with respect to the distances of the BLG. In particular, this interlayer distance widening is more pronounced for the BLG-Li^+^ than for BLG-Li. The BEs, using the minima obtained from the potential energy profiles of this configuration, were not calculated by equation (7), since the convergence of the SCF energy for charged systems, such as slab-Li^+^, cannot be achieved, however, the widening of the equilibrium distance found in the twisted layers, due to the insertion of Li^+^, suggests a greater cation-accommodating capacity of twisted graphene compared to BLG.

This agrees with a greater absorption capacity of the twisted graphene with respect to the non-rotated bilayer as found for the outside configuration. These results pave the way for the study of the selective properties of twisted graphene.

## 4. Conclusions

The interaction of external or intercalated Li and Li^+^ with graphene layers has been the subject of particular attention because of its importance in the applications of graphene layers in LiBs. Graphene layers are often twisted around their parallel plane to obtain twisted graphene with tuneable properties. Thus, in this research, the interactions between Li and Li^+^ with bilayer graphene and twisted bilayer were investigated by a first-principles density functional theory periodic method, considering adsorbates at different symmetry sites.

For the outside configuration, where Li and Li^+^ are located on the single and bilayer graphene, we calculated the potential energy profiles. Then, potential energies of SLG and BLG were fit by Morse-based curves up to 6 Å, and the binding energies were calculated as differences between the energies of the minima and those of the plateaux obtained from the Morse-based curves. We found that the most probable adsorption site for the Li and Li^+^ is the hollow site, both for the SLG and BLG, although we did not notice a large difference with respect to the other sites. The binding energies of some adducts in this configuration were compared with those evaluated by Equation (7), finding a satisfactory agreement among the values obtained through the two approaches.

Based on this finding, we found that the binding energies of Li^+^ systematically increase from bilayer graphene to twisted graphene regardless of twisted angles, while for Li atoms, the BE decreases or remains substantially unchanged depending on the twist angles. This suggests a greater adsorption capacity of twisted graphene towards Li^+^ compared to non-twisted layers, which would increase the adsorption capacity of the twisted graphene-covered anode in LiB.

When the Li or Li^+^ were intercalated between two graphene layers, the equilibrium interlayer distances were again calculated from the potential energy profiles. We found that the equilibrium interlayer distances in the twisted graphene increase compared to the unrotated bilayer with a more significant increase for Li^+^ intercalated layers. This suggests that the twisted graphene can better accommodate the cation in agreement with the above result.

Further investigations are in progress to verify if the found behaviors are maintained when more graphene layers are considered, e.g., four layers.

## Figures and Tables

**Table 1:** Comparison between some key properties of single-layer, bilayer, and few-layer graphene.

Properties	Single Layer	Bilayer	Few-Layer
Electron mobility (cm^2^/Vs)	200,000	∼10^6^	3000–10,000
Thermal conductivity (W/mK)	4840–5300	∼1900	1100
Surface area (m^2^/g)	2630	1628	270–1550
Young’s modulus (TPa)	1.0	0.8	4.6
Optical transparency (%)	∼97.7	∼95	-

**Table 2:** Twisted angle, N_atom_ and L_cell_.

(m, n)	Twist Angle (Deg.)	Atoms/Cell	Cell Size (Å)
(3, 2)	13.17	76	10.72
(2, 1)	21.79	28	6.51
(4, 1)	38.21	84	11.27

**Table 3:** Equilibrium distance and binding energies of Li and Li^+^ on SLG for the H, B, and T sites.

	Site	*d* (Å)	∆*E* (eV)
SLG-Li	B	1.8	0.71
	H	1.6	0.82
	T	1.8	0.57
SLG-Li^+^	B	2.0	0.89
	H	2.0	0.96
	T	2.0	0.87

**Table 4:** Equilibrium distance and binding energies of Li and Li^+^ for BLG in H, B, and T in outside configuration.

	Site	*d* (Å)	∆*E* (eV)
BLG-Li	B	2.0	0.74
	H	1.8	0.96
	T	2.0	0.74
BLG-Li^+^	B	2.0	1.60
	H	1.8	1.75
	T	2.0	1.59

**Table 5:** Equilibrium distance (*d*) and binding energies of Li adsorbed on BLG and t-BLG in the hollow site and outside configuration.

	Twist Angle (Deg.)	*d* (Å)	∆*E* (eV)
BLG-Li	0.00	1.8	9.55
t-BLG-Li	13.17	3.5–6.0	8.16
	21.79	3.5–6.0	8.16
	38.21	1.8	9.82

**Table 6:** Equilibrium distance (*d*) and binding energies of Li^+^ adsorbed on BLG and t-BLG in the hollow site and outside configuration.

	Twist Angle (Deg.)	*d* (Å)	∆*E* (eV)
BLG-Li^+^	0.00	1.8	10.20
t-BLG-Li^+^	13.17	1.8	11.24
	21.79	2.0	-
	38.21	2.0	11.10

**Table 7:** Equilibrium distance (*d*) of Li and Li^+^ adsorbed on bridge and hollow sites of BLG and t-BLG, respectively, for inside configuration (sandwich).

	Twist Angle (Deg.)	*d* (Å)
BLG-Li	0.00	4.0
t-BLG-Li	13.17	4.2
	21.79	-
	38.21	4.1
BLG-Li^+^	0.00	3.4–3.6
t-BLG-Li^+^	13.17	4.2
	21.79	4.2
	38.21	4.0

## Data Availability

Data are contained within the article and Supplementary Materials.

## Supplementary Materials

The following supporting information can be downloaded at: https://www.mdpi.com/article/10.3390/ma17091941/s1, Figure S1: The potential energy profiles of Li (a), (c), (e) and Li^+^ (b), (d), (f) for twist angles of 13.17°, 21.79°, 38.21° respectively, outside configuration; Figure S2: Potential energy profiles of Li (a), (c), (e) and Li^+^ (b), (d), (f) for twist angles of 13.17°, 21.79°, 38.21°, respectively, considering inside configuration (sandwich).

## References

[1] Aversa R., Petrescu V., Apicella A., Petrescu I.T. (2016). The Basic Elements of Life’s. Am. J. Eng. Appl. Sci..

[2] Novoselov K.S., Geim A.K., Morozov S.V., Jiang D., Zhang Y., Dubonos S.V., Grigorieva I.V., Firsov A.A. (2004). Electric Field Effect in Atomically Thin Carbon Films. Science.

[3] Singh S.B., Haskin N., Dastgheib S.A. (2023). Coal-Based Graphene Oxide-like Materials: A Comprehensive Review. Carbon.

[4] Yin Z., Zhu J., He Q., Cao X., Tan C., Chen H., Yan Q., Zhang H. (2014). Graphene-Based Materials for Solar Cell Applications. Adv. Energy Mater..

[5] Choi H.-J., Jung S.-M., Seo J.-M., Chang D.W., Dai L., Baek J.-B. (2012). Graphene for Energy Conversion and Storage in Fuel Cells and Supercapacitors. Nano Energy.

[6] Kucinskis G., Bajars G., Kleperis J. (2013). Graphene in Lithium Ion Battery Cathode Materials: A Review. J. Power Sources.

[7] Lv W., Tang D.-M., He Y.-B., You C.-H., Shi Z.-Q., Chen X.-C., Chen C.-M., Hou P.-X., Liu C., Yang Q.-H. (2009). Low-Temperature Exfoliated Graphenes: Vacuum-Promoted Exfoliation and Electrochemical Energy Storage. ACS Nano.

[8] Wang J., Mu X., Wang X., Wang N., Ma F., Liang W., Sun M. (2018). The Thermal and Thermoelectric Properties of In-Plane C-BN Hybrid Structures and Graphene/h-BN van Der Waals Heterostructures. Mater. Today Phys..

[9] Ohta T., Bostwick A., Seyller T., Horn K., Rotenberg E. (2006). Controlling the Electronic Structure of Bilayer Graphene. Science.

[10] Datta D., Li J., Koratkar N., Shenoy V.B. (2014). Enhanced Lithiation in Defective Graphene. Carbon.

[11] Dervin S., Dionysiou D.D., Pillai S.C. (2016). 2D Nanostructures for Water Purification: Graphene and Beyond. Nanoscale.

[12] Chung C., Kim Y.-K., Shin D., Ryoo S.-R., Hong B.H., Min D.-H. (2013). Biomedical Applications of Graphene and Graphene Oxide. Acc. Chem. Res..

[13] Alhajji E., Zhang F., Alshareef H.N. (2021). Status and Prospects of Laser-Induced Graphene for Battery Applications. Energy Technol..

[14] Zhao W., Shen B., Tao Z., Han Z., Kang K., Watanabe K., Taniguchi T., Mak K.F., Shan J. (2023). Gate-Tunable Heavy Fermions in a Moiré Kondo Lattice. Nature.

[15] Li B., Wei J., Jin C., Si K., Meng L., Wang X., Jia Y., He Q., Zhang P., Wang J. (2023). Twisted Bilayer Graphene Induced by Intercalation. Nano Lett..

[16] He W.-Y., Chu Z.-D., He L. (2013). Chiral Tunneling in a Twisted Graphene Bilayer. Phys. Rev. Lett..

[17] Bistritzer R., MacDonald A.H. (2011). Moiré Bands in Twisted Double-Layer Graphene. Proc. Natl. Acad. Sci. USA.

[18] Uchida K., Furuya S., Iwata J.-I., Oshiyama A. (2014). Atomic Corrugation and Electron Localization Due to Moiré Patterns in Twisted Bilayer Graphenes. Phys. Rev. B.

[19] Scarcello A., Alessandro F., Arias Polanco M., Vacacela Gomez C., Cid Perez D., De Luca G., Curcio E., Caputi L.S. (2021). Evidence of Massless Dirac Fermions in Graphitic Shells Encapsulating Hollow Iron Microparticles. Appl. Surf. Sci..

[20] Moon P., Son Y.-W., Koshino M. (2014). Optical Absorption of Twisted Bilayer Graphene with Interlayer Potential Asymmetry. Phys. Rev. B.

[21] Robledo C.B., Otero M., Luque G., Cámara O., Barraco D., Rojas M.I., Leiva E.P.M. (2014). First-Principles Studies of Lithium Storage in Reduced Graphite Oxide. Electrochim. Acta.

[22] Zhao M., Zhang Z., Shi W., Li Y., Xue C., Hu Y., Ding M., Zhang Z., Liu Z., Fu Y. (2023). Enhanced Copper Anticorrosion from Janus-Doped Bilayer Graphene. Nat. Commun..

[23] Zhou L.-J., Hou Z.F., Wu L.-M. (2012). First-Principles Study of Lithium Adsorption and Diffusion on Graphene with Point Defects. J. Phys. Chem. C.

[24] Larson D.T., Carr S., Tritsaris G.A., Kaxiras E. (2020). Effects of Lithium Intercalation in Twisted Bilayer Graphene. Phys. Rev. B.

[25] Aprà E., Bylaska E.J., de Jong W.A., Govind N., Kowalski K., Straatsma T.P., Valiev M., van Dam H.J.J., Alexeev Y., Anchell J. (2020). NWChem: Past, Present, and Future. J. Chem. Phys..

[26] Valiev M., Bylaska E.J., Govind N., Kowalski K., Straatsma T.P., Van Dam H.J.J., Wang D., Nieplocha J., Apra E., Windus T.L. (2010). NWChem: A Comprehensive and Scalable Open-Source Solution for Large Scale Molecular Simulations. Comput. Phys. Commun..

[27] White J.A., Bird D.M. (1994). Implementation of Gradient-Corrected Exchange-Correlation Potentials in Car-Parrinello Total-Energy Calculations. Phys. Rev. B.

[28] Hamann D.R., Schlüter M., Chiang C. (1979). Norm-Conserving Pseudopotentials. Phys. Rev. Lett..

[29] Morse P.M. (1929). Diatomic Molecules According to the Wave Mechanics. II. Vibrational Levels. Phys. Rev..

[30] Tang B., Wang Y.-T., Peng X.-L., Zhang L.-H., Jia C.-S. (2020). Efficient Predictions of Gibbs Free Energy for the Gases CO, BF, and Gaseous BBr. J. Mol. Struct..

