# Supplementary material for: Unraveling the Interactions between Lithium and Twisted Graphene
Source: Materials (Basel). 2024 Apr 23;17(9):1941. doi: 10.3390/ma17091941 (PMC11084250; doi:10.3390/ma17091941)
Supplement: Supplementary file 1 [file materials-17-01941-s001.zip › materials-2948511-supplementary.pdf]

Supplementary Materials

Unraveling the Interactions between Lithium and Twisted Graphene

Maximo Ramírez [1], Giorgio De Luca [2,*] and Lorenzo Caputi [3]

1 Independent Researcher, 87036 Rende, Italy
2 Research Institute on Membrane Technology (ITM-CNR), c/o University of Calabria, 87036 Rende, Italy
3 Surface Nanoscience Group, Department of Physics, University of Calabria, 87036 Rende, Italy
* Correspondence: g.deluca@itm.cnr.it

The potential energy profiles of Li and Li^{+} referring to t-BLG were evaluated for slab-Li/Li+ distances up to 6 Å considering three symmetry adsorption sites (Top, Hollow, Bridge) and outside configuration. The potential energies plots are shown in Figure1S

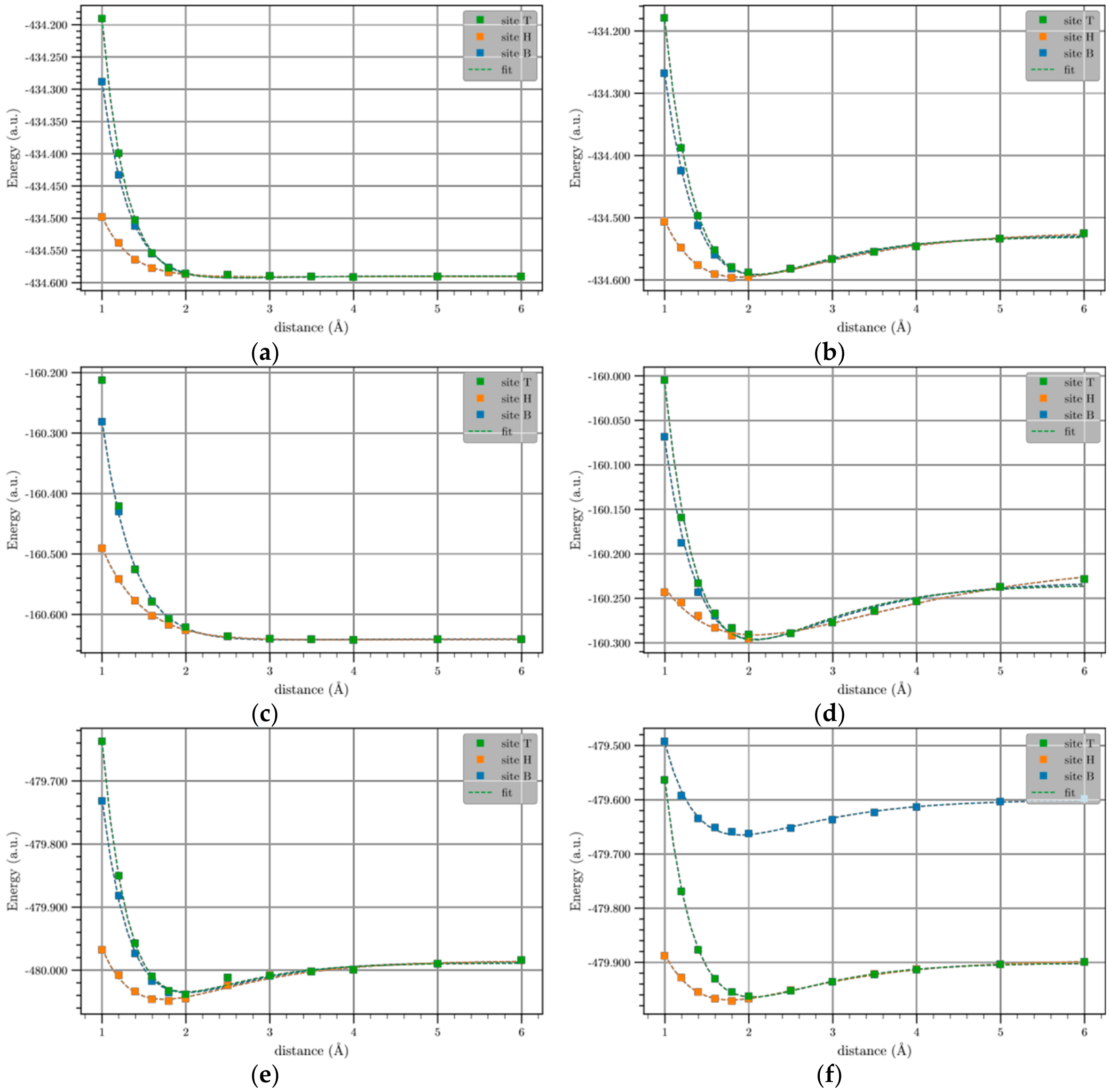

Figure S1. The potential energy profiles of Li (a), (c), (e) and Li^{+} (b), (d), (f) for twist angles of 13.17°, 21.79°, 38.21° respectively, outside configuration.

The potential energy profiles of Li and Li^+ referring to t-BLG were evaluated for interlayer distances up to 6 Å considering three adsorption sites and inside configuration. The potential energies plots are shown in Figure S2

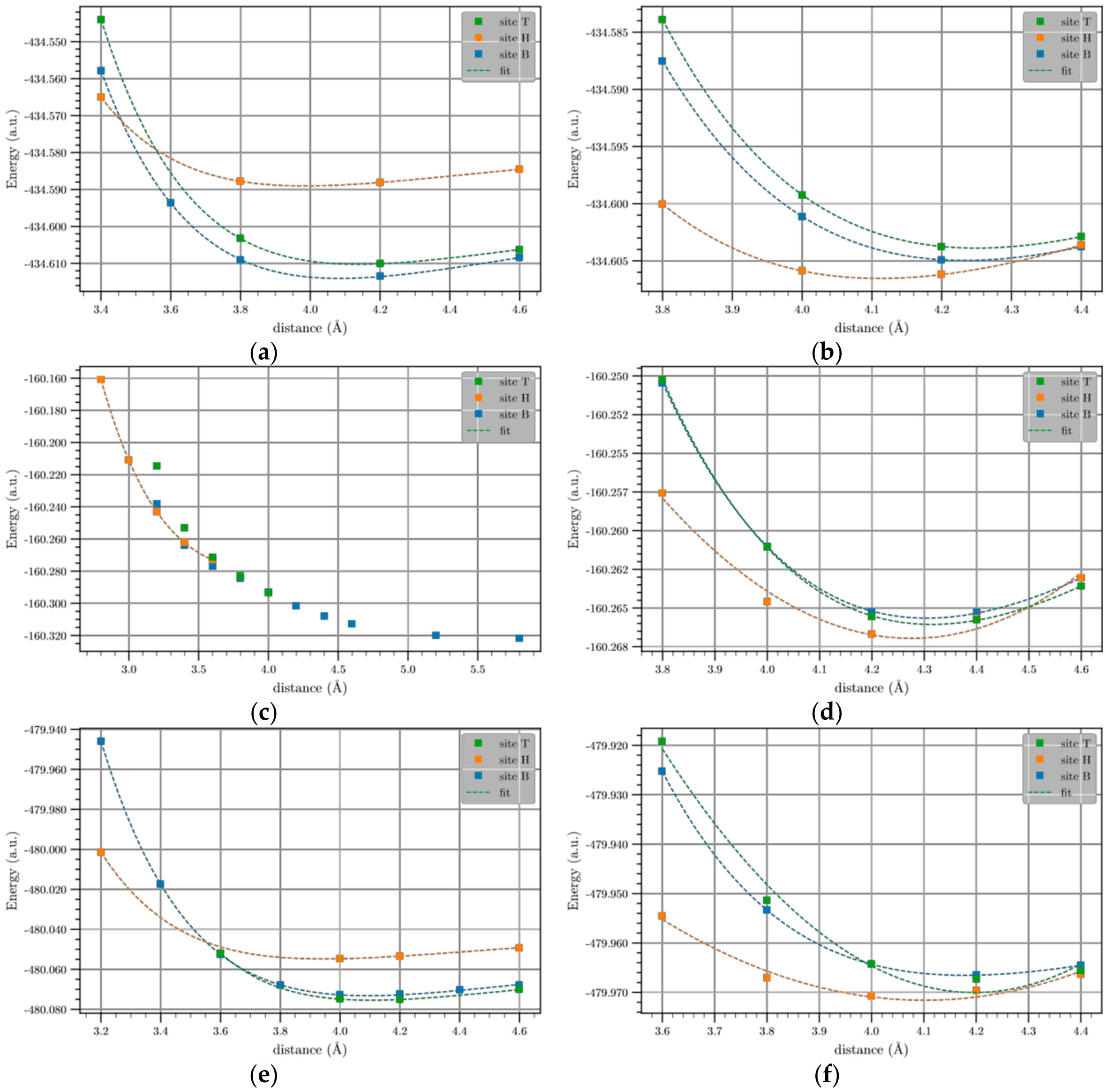

Figure S2. Potential energy profiles of Li (a), (c), (e) and Li^+ (b), (d), (f) for twist angles of 13.17°, 21.79°, 38.21°, respectively, considering inside configuration (sandwich).
